# First Detection of Anti-*Besnoitia* spp. Antibodies in Equids in Israel and the Palestinian Authority

**DOI:** 10.3390/microorganisms11040929

**Published:** 2023-04-03

**Authors:** Noa Berman, Sharon Tirosh-Levy, Amir Steinman, Avital Minderigiu, Elena Blinder, Monica Leszkowicz Mazuz

**Affiliations:** 1 Koret School of Veterinary Medicine, The Robert H. Smith Faculty of Agriculture, Food and Environment, The Hebrew University of Jerusalem, Rehovot 7610001, Israel; 2 Division of Parasitology, Kimron Veterinary Institute, Bet Dagan 5025004, Israel

**Keywords:** *Besnoitia benneti*, horses, donkeys, IFAT, serology, Israel

## Abstract

*Besnoitia* is a tissue cyst forming coccidia, which affects multiple host species worldwide. Equine besnoitiosis is characterized mainly by generalized skin lesions and cysts in the scleral conjunctiva. Recent reports revealed exposure to *Besnoitia* in equines in Europe and the United States. However, the exposure to *Besnoitia* spp. in the Israeli equine population was never investigated. The aim of this study was to evaluate the seroprevalence and associated risk factors for besnoitiosis in equids in Israel. A cross-sectional serosurvey was performed using serum samples of apparently healthy horses (*n* = 347), donkeys (*n* = 98), and mules (*n* = 6), and exposure to *Besnoitia* spp. was determined by an immunofluorescent antibody test (IFAT). Anti-*Besnoitia* spp. antibodies were detected in 17.7% equids, 6.9% horses, 33.3% mules, and 55.1% donkeys. The seroprevalence in donkeys was significantly higher than in horses (*p* < 0.001). A significant association between the geographic location and seropositivity was found both in horses and donkeys, which was significantly higher (*p* = 0.004) in horses sampled in southern Israel, and donkeys sampled in Israel versus the Palestinian Authority (*p* < 0.001). This is the first serosurvey of *Besnoitia* infection in equines in Israel, and the results are consistent with reports from Europe. The clinical significance of equine besnoitiosis should be further investigated.

## 1. Introduction

*Besnoitia* is an apicomplexan protozoan parasite and a tissue cyst forming coccidia. The genus *Besnoitia* belongs to the family Sarcocystidae and to the subfamily Toxoplasmatinae, along with the closely related protozoa *Neospora caninum* and *Toxoplasma gondii* [1]. Several *Besnoitia* species were described up to date. *Besnoitia besnoiti* is the species associated with bovine besnoitiosis, leading to extensive economical and animal welfare consequences attributed to mortality, decreased milk production, and sterility of bulls [2,3]. *Besnoitia bennetti* is associated with equine besnoitiosis, which was reported in horses and donkeys in Africa, Asia, and more recently, in Europe and the United States (US) [4,5,6,7,8,9]. The emergence and spread of both bovine and equine besnoitiosis in Europe and the US over the last two decades led to increasing awareness and research efforts regarding this poorly understood species.

The life cycle for all *Besnoitia* species is not fully elucidated. It is suggested to have a facultative two-host (heteroxenous) life cycle, with carnivorous species acting as the definitive host for several species and other mammals as intermediate hosts [10]. Feline species were identified as the definitive hosts for several *Besnoitia* species; however, the definitive host for *B. besnoiti* and *B. bennetti* were never identified [9,10].

The transmission of *Besnoitia* spp. is poorly understood. Three routes of horizontal transmission were suggested to be significant: (1) direct transmission via contact with lacerations of infected animals, through the naso-pharyngeal route or through natural mating [11,12], (2) indirect transmission by blood sucking arthropods [3], and (3) digestion of oocysts excreted by the definitive host [13]. The significance of each of these mechanisms in the transmission of different *Besnoitia* species is still unknown, since the definitive host and competent arthropod vectors were not identified. Vertical transmission is an important mode of transmission for the closely related protozoan *Toxoplasma gondii* and the major mode of transmission for *Neospora* spp. however, it was never documented for *Besnoitia* spp. [14]. Identifying the major routes of transmission of besnoitiosis is important for the understanding of the epidemiology of this disease and evaluating the risk of future spread. The mode of transmission in equine infection still remains unknown [6].

The pathophysiology and clinical disease of besnoitiosis in intermediate hosts was mainly investigated in cattle. The disease in cattle has an acute stage and a chronic stage. The acute stage is characterized by rapid proliferation of tachyzoites in macrophages, fibroblasts, and endothelial cells. This stage clinically lasts three to ten days and manifests by hyperthermia, with a variety of additional symptoms, which may affect productivity and reproduction [2,14]. The chronic stage is characterized by the formation of tissue cysts up to 0.5 mm in diameter. In contrast to other cyst-forming coccidia species, which form tissue cysts in muscle and brain tissues, *B. besnoiti* tissue cysts are mostly found in mesenchymal tissues in the dermis, sclera, and mucosa [15]. These peripheral tissue cysts may allow for direct or mechanical transmission. Clinically, this stage is characterized by chronic scleroderma (“elephant skin”), a progressive thickening, and wrinkling of the skin, accompanied by additional focal and systemic signs [2,14]. The severity of the skin lesions is directly related to the number of cysts and the parasite load [16]. Death may occur in both the acute and chronic stages of the disease, although mortality is rare [2,14]. The economic effect of bovine besnoitiosis is attributed to case fatality, but mostly to decreased milk production, decreased fertility in bulls, and poor leather quality [14].

Equine besnoitiosis is characterized by multifocal white pinpoint miliary parasitic cysts in the skin, both on the face and torso (in the nares, on the pinnae, and on the limbs and perineum). As in cattle, the pathognomonic feature of the disease is the development of parasitic cysts within the sclera and conjunctiva of the eye (scleral pearls). With the progression of disease, infected animals develop poor hair coat and skin lesions consisting of alopecia, hypotrichosis, hyperpigmentation, thickening, and crusting, involving the face, muzzle, eyes, ears, neck, flanks, legs, and perineum. Donkeys appear to be more clinically affected by besnoitiosis than horses, with more clinical reports of donkeys rather than horses [6,9,17,18]. Some infected animals remain otherwise healthy, while others become cachectic and debilitated [6,8].

Diagnosis of infected animals can be based on clinical signs, in combination with cytology and histopathology from suspected infected tissues [3,11,19,20,21,22]. Identification of the parasite using molecular tools, including conventional and real-time polymerase chain reaction (PCR) is possible, although not always practical, since the detection of parasite DNA relies on the accurate sampling of infected tissue. Therefore, these methods are mostly used for post-mortem evaluation or biopsies from suspected dermal cysts [21]. Hence, in many cases, diagnosis is made by serological approach. Several serological tests, such as the immunofluorescent antibodies test (IFAT), enzyme-linked immunosorbent assay (ELISA), and Western blot (WB), are available for detection of specific antibodies, which develops 15–16 days post infection. All performed well during the chronic stage of the disease [20,23,24], but at the same time all show low sensitivity during the acute stages, as detectable antibody levels were yet developed [14].

The epidemiology of besnoitiosis in horses and donkeys around the world evolved from a sporadic disease, mainly in Africa, to an emerging disease in the US [6,8,17], and many European countries, including Spain [25], Italy [18], Portugal [26], the United Kingdom (UK) [7], and Belgium [27]. In recent years, there is increasing awareness to besnoitiosis in equines, with increasing clinical descriptions (especially from donkeys), and serological reports from horses and donkeys worldwide [22,23,24,25,26].

Besnoitiosis in Israel was reported in cattle since the 1960’s [28], with prevalence rates of 64.4–66.9% in beef cattle [29,30]. Despite the routine use of the live tachyzoite vaccine on stud bulls since the 1990’s, there are still sporadic reports of clinical besnoitiosis in cattle in Israel annually (https://www.gov.il/BlobFolder/reports/annual-report-veterinary-services/he/animals_health_doch_shnati_2019.pdf, accessed on 30 December 2022). However, data regarding *Besnoitia* spp. exposure among equids were never reported in Israel. Although equine and bovine infection is attributed to different *Besnoitia* species, both *B. besnoiti* and *B. benetti* were reported from similar geographical areas [14]. Both these parasites are considered endemic in Africa and Asia (mostly *B. besnoiti*) and emerging in the US (mostly *B. benetti*) and Europe [14].

The aim of this study was to estimate the seroprevalence of *Besnoitia* spp. in equids (horses, donkeys, and mules) in Israel and to determine the risk factors associated with seropositivity.

## 2. Materials and Methods

### 2.1. Sample Collection for Serological Survey

A sample size for a cross-sectional study was calculated using the statistical software Winpepi (Version 11.43), with a confidence level of 95% and an acceptable difference of 5%, assuming an expected seroprevalence of 7% in horses [31] and 22% in donkeys [31,32]. The required samples size was 189 horses and 77 donkeys.

Serum samples were collected from 347 apparently healthy horses in 30 farms throughout Israel during 2018. Farms were chosen to represent the distribution of the horse population in Israel. Since no data are available for estimating the distribution of donkeys in Israel, donkeys were sampled at two donkey shelters in Israel that receive donkeys from different locations, and at three locations in the Palestinian Authority (PA), to which working animals were brought to receive veterinary care given through a humanitarian association. A total of 98 donkeys were sampled. Six mules were also sampled and included in this study.

Blood was extracted from the jugular vein of each animal into sterile vacuum serum separation tubes. Serum was separated after centrifugation (3000 rpm for 10 min) and kept at −20 °C until use. During sampling, data regarding the characteristics of each animal were recorded (age, breed, sex, housing management, and the farm’s geographic location). The geographical location of each farm was defined as “north”, “center”, or “south”, with the center defined between latitude 32.47 and 31.81.

All samples were obtained with the owner’s permission, and the study was approved by the Hebrew University Ethics Committee (KSVM-VTH/23_2014, HU-NER-2020-055-A).

### 2.2. Serological Screening Using Immunofluorescent Antibody Test (IFAT)

All sera were tested for *Besnoitia* spp. exposure by using IFAT, as previously described [33,34]. In brief, *Besnoitia* antigen slides were prepared from *B. besnoiti* isolated from a naturally infected bull [34]. Dilutions of sera were performed in bovine serum albumin (BSA) buffer 1% at an initial screening dilution of 1:64. All samples that showed fluorescence at the 1:64 dilution were further diluted at a 1:4 ratio to the endpoint titer. The highest dilution of serum exhibiting fluorescence of the whole *Besnoitia* organism was considered as the endpoint titer. A titer of 1:64 was considered as a cut-off for positivity for *Besnoitia* exposure [33], and the results were also interpreted using a cutoff titer of 1:156. The diluted sera were applied to the slide antigens and incubated for 30 min at 37 °C in a humid chamber. The slides were washed in a Carbonate buffer (pH 9) for 10 min, dried, and anti-horse Ig-G conjugate with fluorescein (SIGMA^®^ Israel) at 1:80 dilution with BSA buffer was added. The slides were then incubated at 37 °C for 30 min, washed as described above, mounted under coverslips with glycerol/carbonate buffer (1:1), and examined under a fluorescence microscope. In the absence of equid positive and negative controls, control samples from cattle previously tested for the presence of anti-*Besnoitia* antibodies were initially used, until a positive and negative for both horse and donkey samples were identified. In addition to the negative serum control, phosphate-buffered saline (PBS) without serum was also added as a second negative control in each run [33].

### 2.3. Additional Tests of Positive Samples for Other Cystogenic Coccidia

Samples of horses and donkeys that tested positive for *Besnoitis* spp. were additionally tested for the presence of anti-*Neospora* spp. and anti-*Toxoplasma gondii* antibodies, to evaluate the presence of cross-reactivity between closely related parasites. Serological examinations were performed by IFAT, as previously described [35,36,37]. The cut-off titer for *T. gondii* was set as 1:64, and for *Neospora* spp. was set as 1:50 [38,39,40].

### 2.4. Statistical Analysis

*Besnoitia* spp. seroprevalence was calculated as the percentage of seropositive animals within the study population. Associations between potential risk factors and *Besnoitia* seropositivity were analyzed in horses and in donkeys separately and together. Mules were not included in the statistical analysis, due to the small sample size. The association between continuous parameters and seropositivity was evaluated using a *t*-test, while categorial parameters were analyzed using the χ^2^ test or Fisher’s exact test and odds ratios (OR) with 95% confidence intervals (95% CI) were calculated. All factors that were found to be significantly associated with *Besnoitia* seropositivity were included in a multivariable generalized estimating equation (GEE) using the logit link function, with each animal defined as subject and the farm as within-subject effect. Fisher’s exact test was used to compare the seroprevalence between horses, donkeys, and mules. Statistical significance was set at *p* < 0.05. The analysis was performed using the SPSS 22.0^®^ and Win Pepi 11.43^®^ statistical software.

## 3. Results

### 3.1. Study Population

**Figure 1:**
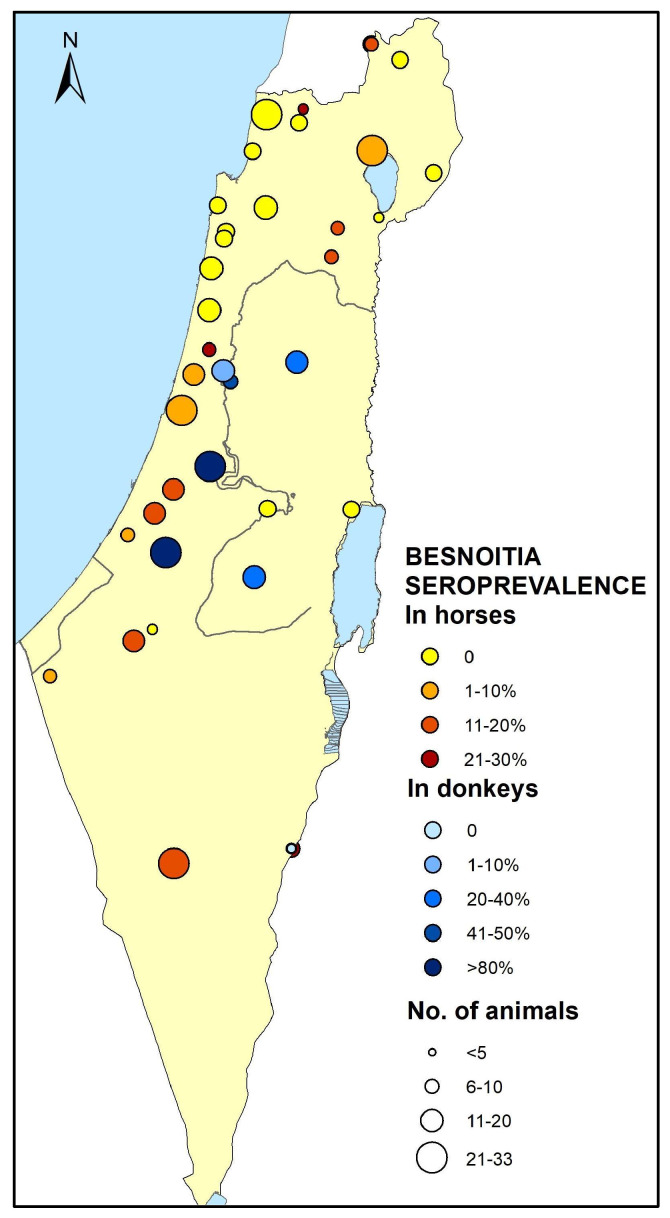
The geographical distribution of the study population, and *Besnoitia* seroprevalence at each farm. Horse farms appear in red and donkey farms in blue. The size of each circle represents the number of animals sampled, while the intensity of the color reflects *Besnoitia* seroprevalence at each farm.

The study population comprised 347 horses from 30 different farms throughout Israel. Between four and 33 horses were sampled at each farm. The geographical distribution of the farms included 16 farms in the north (*n* = 160 horses, 46.1%), six farms in the center (*n* = 97 horses, 27.9%), and eight farms in the south (*n* = 90 horses, 25.9%) (Figure 1). Most of the horses were geldings (*n* = 171, 49.3%) followed by mares (*n* = 166, 47.8%) and a few were stallions (*n* = 10, 2.9%). The majority of horses were mixed-bred (*n* = 163, 46.9%), while the others were of 16 different breeds, including Quarter horses (*n* = 69, 19.9%), Arabian horses (*n* = 47, 13.5%), Warmbloods (*n* = 17, 4.9%), Ponies (*n* = 17, 4.9%), Tennessee Walking horses (*n* = 11, 3.2%), and various other breeds (Paint horse, Thoroughbred, Appaloosa, Missouri Foxtrot, Friesian, Andalusian, Haflinger, Shire, Miniature, each *n* = <10). The mean age of the horses was 11.6 years (standard deviation (SD) = 6.1) and the median age was 11 years (inter quartile range (IQR) = 8), the youngest horse was six months old and the oldest was 47 years old.

A total of 98 donkeys were sampled at two geographic areas: Israel (*n* = 49) and the Palestinian Authority (PA, *n* = 49). Samples from Israel came from two donkey shelters located in the central region (*n* = 25, and *n* = 23) and from one privately owned donkey in the south. In the PA, donkeys were sampled at four locations (*n* = 14, *n* = 13, *n* = 13, and *n* = 9), in a similar geographical area to the donkeys sampled in Israel (Figure 1). Due to the nature of the sampled population, the data available regarding each donkey were limited. Most of the donkeys were males (*n* = 60, 61.2%). Data regarding the age were available for 70 of the donkeys. The mean age was 7.6 years (SD = 5.1) and the median age was 7 years (IQR = 6), the youngest donkey was 4 months old, and the oldest was 25 years old.

Six mules were sampled at one of the horse farms (in Israel, *n* = 1) and at two of the donkey sampling locations (both in the PA, *n* = 5). The mean age of the mules was 14 years (SD = 6.9) and the median age was 12 years (IQR = 12), the youngest mule was 7 years old, and the oldest was 25 years old.

### 3.2. Besnoitia *spp.* Seroprevalence

Anti-*Besnoitia* spp. antibodies were detected in 80 out of 451 equids (17.7% 95% CI: 14.3–21.6). The seroprevalence in horses was 6.9% (24/347, 95% CI: 4.5–10.1), 55.1% in donkeys (54/98, 95% CI: 44.7–65.2), and 33.3% in mules (2/6, 95% CI: 4.3–77.7). The seroprevalence in donkeys was significantly higher than that in horses (OR = 15.86, 95% CI: 8.65–29.23, *p* < 0.001).

At least one seropositive horse was found in 15 of the 30 horse farms (50%). The seroprevalence in positive farms ranged between 4.5% and 28.5%. Seropositive donkeys were identified at all sampling locations, except for the single, privately owned donkey, which was seronegative. The seroprevalence at every sampling location ranged between 7.7% and 88% (Figure 1).

**Figure 2:**
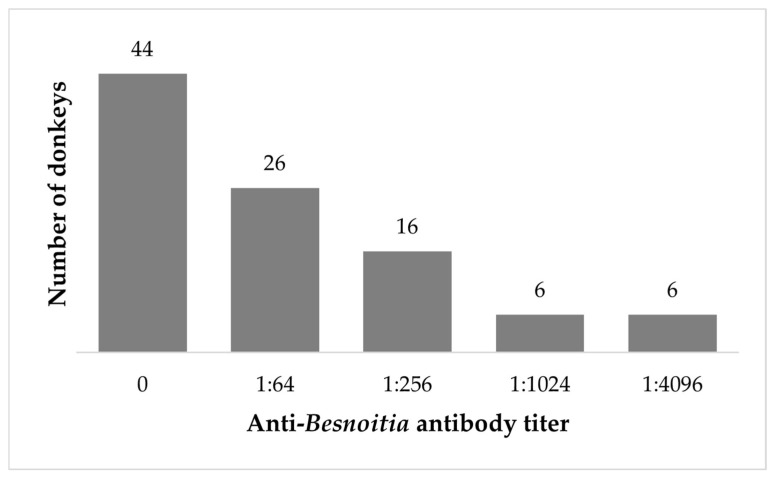
Anti-*Besnoitia* spp. antibodies titer in positive donkeys. The numbers above the bars represent the number of donkeys that were seropositive in the end point titer.

All seropositive horses had an end point titer of 1:64. The serological titers of positive donkeys ranged between 1:64 and 1:4096 (Figure 2). Nearly half of the seropositive donkeys had an antibody titer of 1:64 (26/54 seropositive donkeys, 48.1%), while the median titer in the seropositive group was 1:256 (IQR = 4032). The two seropositive mules had antibody titers of 1:64 and 1:256.

When selecting a higher cutoff titer for seropositivity (1:256), 29 out of 451 equids were seropositive for *Besnoitia* (6.4%, 95% CI: 4.3–9.1). These comprised of 28 seropositive donkeys (28/98, 28.6%, 95% CI: 19.9–38.6) and one seropositive mule (1/6, 16.7%, 95% CI: 0.4–64.1). None of the horses were seropositive when using the higher cutoff.

### 3.3. Co-Exposure to Other Cystogenic Coccidia

Out of the twenty-four *Besnoitia* seropositive horses, six (25%) tested positive solely to *Besnoitia* spp., eight (33%) tested seropositive to *Neospora* spp., one (4%) tested seropositive to *Toxoplasma gondii*, and nine (37%) horses were seropositive to all three parasites.

Out of 54 *Besnoitia* seropositive donkeys, all were positive to *Toxoplasma gondii,* and 44 (81.4%) were seropositive to *Neospora* spp.

Out of the 28 *Besnoitia* seropositive donkeys, using a cutoff titer of 1:256, all were also positive to *Toxoplasma gondii,* and 24 (85.7%) were also seropositive to *Neospora* spp.

### 3.4. Risk Factors for Besnoitia Seropositivity

*Besnoitia* seroprevalence among horses in the north, center, and south of Israel was 3.1%, 7.2%, and 14.2%, respectively, and the geographical area was significantly associated with exposure (*p* = 0.004, Table 1, Figure 1). The seroprevalence in horses from southern Israel was significantly higher than in the north or center (OR = 3.4, 95% CI: 1.36–8.5, *p* = 0.004). The mean age of seropositive and seronegative horses was 12.7 years (SD = 5.4) and 11.5 years (SD = 6.1), respectively, which did not differ significantly (*p* = 0.331).

Similar to the result in horses, a significant association was found between *Besnoitia* spp. seropositivity in donkeys and the geographic location. Donkeys sampled in Israel had higher prevalence than donkeys sampled in the PA (*p* < 0.001, OR = 14.2, 95% CI: 4.8–43.5, Table 1). Age was found to be a significant risk factor for donkeys. The mean age of seropositive donkeys (9.2 years, SD = 4.67) was significantly higher than that of seronegative donkeys (6.4 years, SD = 4.67, *p* < 0.05). Both age and the geographic location were included in the multivariable model. Only the geographic location remained statistically significant (OR = 10.3, 95% CI: 3.2–33.1, *p* < 0.001).

When horses and donkeys were analyzed together, the animal species and geographical area were found to be significantly associated with seropositivity in the univar-iable analysis (*p* < 0.001, Table 1). Donkeys had higher seropositivity than horses (OR = 15.86, 95% CI: 8.65–29.23, *p* < 0.001). In this analysis, the geographical area was defined for donkeys according to the latitude, similarly to the horses. Animals sampled in the south or center had higher seropositivity than in the north (OR = 10.7, 95% CI: 4.2–34.5). However, there were no donkeys sampled in the north. The mean age of seropositive and seronegative animals was 10.9 years (SD = 5.3) and 10.9 years (SD = 6.2), respectively, which did not differ significantly (*p* = 0.992). Both the animal species (*p* < 0.001) and the geographical area (*p* = 0.006) remained significantly associated with seropositivity in the multivariable model. Donkeys had significantly higher seroprevalence than horses (OR = 12, 95% CI: 6.3–22.7, *p* < 0.001). Animals sampled in the south had higher seroprevalence than in the north (OR = 4.7, 95% CI: 1.7–13.3, *p* = 0.004), but not in the center (*p* = 0.1). The interaction between animal species and geographical area was not included in the multivariable model, since no donkeys were sampled in the north.

When analyzing the results using a higher cutoff titer of 1:256, none of the horses were seropositive. All positive donkeys were sampled in Israel, and none in the PA (*p* < 0.001, Table 1). The mean age did not differ significantly between seropositive donkeys (7.9 years, SD = 4.2) and seronegative donkeys (7.5 years, SD = 5.3, *p* = 0.843). Since all positive animals were donkeys, and since no donkeys were sampled in the north, a multivariable model could not be fitted properly to the data.

## 4. Discussion

This study was the first to evaluate the exposure to *Besnoitia* spp. in equids in Israel. Israel is situated in the Middle East, between Europe and Africa, near the Mediterranean Sea. Due to its unique location, it may be an indicator for the spread of infectious diseases between Africa and Europe. Besnoitiosis was reported in equines in Africa [41], and more recently in Europe [5,7,31,32]; however, it was never investigated in equines in the Middle East. The results of this study reveal a seroprevalence (using a cutoff titer of 1:64) of 17.7% in the equine population in Israel, with seropositivity of 6.9% in horses, 33.3% in mules, and 55.1% in donkeys. Using a higher cutoff titer of 1:256, in order to increase the stringency of the IFAT test, revealed a seroprevalence of 6.4% in the equine population; however, seropositivity was detected in donkeys (28.6%) and mules (16.7%), but not in horses.

Our results are slightly higher than other reports from Mediterranean countries in Europe. In Spain, the overall besnoitia seroprevalence in equids was 7.1%, including 2.9% (16/553) in horses, 15.3% (13/85) in donkeys, and 26.5% (22/83) in mules being seropositive [31]. Lower seroprevalence was reported in Portugal with 0.3% (1/385) of horses being seropositive [26], and in Italy with overall equine seroprevalence of 2.1% (2/268 horses and 4/18 donkeys) [32]. The differences in seropositivity between studies could reflect actual differences in the distribution of besnoitiosis, but may also be affected from differences in the study populations and in the methods used. Different serological tests and varying cutoff titers may result in changes in sensitivity and specificity. Since no “gold standard” or widely recommended diagnostic method was established, a comparison between reports should be conducted with caution.

The significantly higher seroprevalence found in donkeys may be attributed to different husbandry and management of donkeys and horses. Most of the donkeys were sampled in shelter farms, which means that these donkeys were potentially previously kept in poor conditions. Although the main route of transmission of besnoitiosis in equines is not fully elucidated, poor management is likely to contribute to increased horizontal transmission, as in other cyst-forming coccidia species. Higher seroprevalence in donkeys, compared to horses, was also reported in Italy [32] and Spain [31,32], which may suggest similar differences in management or a difference in susceptibility between these species.

In addition to the higher seroprevalence, the antibody titers of positive donkeys were higher than of seropositive horses. While all positive horses had low antibody titer (1:64, which was the cutoff for positivity), the antibody titers of donkeys ranged between 1:64 and 1:4096. Clinical besnoitiosis was reported mostly in donkeys rather than in horses [6,9,18,42,43]. Clinical besnoitiosis was never reported in equines in Israel. Although all animals sampled in this study were apparently healthy, the differences in antibody titers may be attributed to the fact that in clinical infection, more parasitic activity is present, since there is a direct correlation between the severity of clinical disease and parasitic load [14]. Both the higher seroprevalence and higher antibody titers in donkeys may be attributed to the differences in management, biosecurity, and the use of arthropod repellent in donkeys compared with horses, as this might favor transmission of *Besnoitia* by arthropods [31], or increase susceptibility. Differences in susceptibility for *Besnoitia* infection among cattle breeds were observed [44,45]. Hence, similar differences between horses and donkeys may occur and need to be further investigated. Additionally, as this work presents a serological survey in a determined point and no progressive or retrospective evaluation of the clinical and serological status was conducted, it is not possible to conclude if the donkeys with high titers of antibodies were recently infected or if they keep high titers from a chronic or past infection.

The main risk factor found to be associated with seropositivity in both horses and donkeys was the geographical area. In horses, higher seroprevalence was observed in the southern region of Israel. This may be attributed to the warmer climate conditions in this area that may be beneficial to some arthropods transmitting the parasite [3]. The fact that this difference was also significant when horses were analyzed separately (from donkeys) strengthens the conclusion that this change is not due to a confounding effect of animal species or management system.

In donkeys, higher seroprevalence was observed in the two shelter farms sampled in Israel. One of these two farms was located in southern Israel, while the other was in the center. However, since the donkeys sampled in the PA were from similar geographical locations in this case, the higher seroprevalence may be related to husbandry conditions rather than climate. The two shelter farms in Israel receive donkeys that are found or confiscated, with a history of poor management. In contrast, the donkeys sampled in the PA were brought for a veterinary examination by their owner, and therefore, were potentially better kept prior to sampling.

Besnoitiosis was first reported in Israel in cattle in 1960 with high prevalence in beef cattle herds in extensive management [29,30]. Since the use of live tachyzoites vaccine, reports of clinically infected animals decreased, and only a few sporadic cases in cattle are diagnosed annually (https://www.gov.il/BlobFolder/reports/annual-report-veterinary-services/he/animals_health_doch_shnati_2019.pdf, accessed on 30 December 2022). Physical proximity to infected cattle or wildlife was not tested as a risk factor in this study, due to the challenge in determining the distance from free ranging animals in a large grazing area. Moreover, no evidence of transmission of *Besnoitia* species between equids to cattle was reported [11,46,47]. Proximity to wildlife also was not investigated, as a serosurvey of *Besnoitia* spp. exposure in wildlife in Israel did not yield positive results [28], suggesting that wild animals probably do not play an important role in the circulation of *Besnoitia* in this area.

In this study, exposure to *Besnoitia* spp. was evaluated by serology. Seropositivity suggests exposure and does not necessarily imply current infection. The serological test used in this study (IFAT) was validated for besnoitiosis and was successfully used for cattle and other species diagnostics [26,28,33]. The sensitivity and specificity of the IFAT for detecting besnoitiosis in donkeys, using *B. besnoiti* slides, is high and reached 88% and 96% in a screening performed in the United States of America [9]. Serodiagnosis is considered to be an effective method for the diagnosis of *Besnoitia* in equines, since it is specific, easy to operate, and less invasive than histopathology [9]. Since there were no clinically affected animals in this study, no biopsies or histopathological examinations were performed to demonstrate current infection.

The exact *Besnoitia* species that infected the equids in this study is uncertain. The antigen used in the IFAT slides was *B. besnoiti* tachyzoites from cattle [34], yet cross-reactivity between *Besnoitia* species was described [48]. It is known that equine besoitiosis is mostly attributed to *B. benneti*, infection [9]. However, in order to identify the infecting *Besoitia* spp., a different diagnostic method should be applied, such as PCR or DNA sequencing. Since these methods can only be applied to clinical samples from infected tissues [7], it was not possible to apply them in this study. Further research is needed to identify the species of *Besnoitia* infecting equids in Israel.

Serological cross-reactivity between *Besnoitia* and other cystogenic coccidia species was reported. In particular, some *Neospora*-positive cattle may have false-positive results when tested for *B. besnoiti* exposure [20,24,49,50,51]. Additionally, the serological cutoff for positivity was not determined for horses, which makes it more difficult to interpret the results and to compare them to other studies. In order to address this problem, some studies investigating besnoitiosis in horses used a second assay, such as Western blot, to confirm seropositivity detected by IFAT or ELISA [26,31,32]. The choice of a correct diagnostic test is critical for the reliability and interpretation of the results. Thus, IFAT was chosen for use in this study, as it is widely used and is a well-established method for diagnosis of several cystogenic parasites and has high sensitivity and specificity in the detection of several parasitic diseases [9,34,35,36,37,38,52,53]. Still, to improve the specificity and better comprehend the cross-relativity with closely related parasites, all seropositive samples were also tested for neosporosis and toxoplasmosis. Co-exposure to *Neospora* spp. *Toxoplasma gondii* or all three parasites was found both in horses and in donkeys. The fact that some of the horses were only positive for *Besnoitia* strengthens the likelihood that the IFAT results are specific. The fact that all positive donkeys were also positive to *Toxoplasma gondii* most likely reflects the high seroprevalence of toxoplasmosis within the donkey population, as described in a previous study that used the same study population [35]. In order to better interpret the serological results of equid populations and to determine which serological test is the best for screening equid besnoitiosis, further research aiming to verify the cutoff titer for seropositivity related to clinical infection using different serological methods, and the relationship between parasite load, antibody titer, and clinical outcome is strongly desired.

The relatively high co-exposure rates of these closely related, cyst-forming parasites may suggest common risk factors for infection. The seroprevalence of toxoplasmosis and neosporosis in the same donkey population was very high (94% and 70%, respectively, [35]) and therefore, increased the chance for co-infection. Since almost all donkeys tested seropositive to *Toxoplasma gondii*, no cases of single *Besnoitia* exposure were identified. Interestingly, while *Toxoplasma* was the main parasite identified in donkeys, it was only identified in one of the 24 seropositive horses. In horses, most co-exposures were of *Besnoitia* and *Neospora.* A recent study, which was based on the same serum samples, found 24% seroprevalence of neosporosis in horses in Israel [36]. Therefore, the rate (71%, 95% CI: 48.9–87.4) of co-exposure found in horses was higher than expected and suggests an increased risk of infection with both parasites. Further research is needed to elucidate the interactions between the various Apicomplexan parasites and their clinical significance in equids.

In the performance and interpretation of the obtained results, we were faced with two principal limitations that should be taken in account in further studies. First, we made a one-point sero-survey; thus, no conclusions concerning the dynamics of antibodies or the clinical significance of the antibody titers could be reached. Second, as the amount of the serum was limited and we performed several IFAT assays, evaluation of the cross-reactivity of other serological tests, such as ELISA, agglutination test, or Western blot, which require larger serum volumes, could not be performed.

## 5. Conclusions

This is the first serosurvey of *Besnoitia* spp. exposure in equids in Israel. This study revealed relatively high seroprevalence in equids (17.7%) with seropositivity observed in 6.9% of the horses, 33.3% of the mules, and 55.1% of the donkeys. The risk factor identified to be significantly associated with seropositivity was the geographical area, with higher seroprevalence in horses from southern Israel and in donkeys in Israel rather than the Palestinian Authority. High rates of co-exposure with other cyst-forming coccidia species were observed, suggesting potential common risk factors. The clinical significance of *Besnoitia* infection in equids in Israel is not yet determined, and should be further investigated.

## Figures and Tables

**Table 1:** The association of various risk factors and the *Besnoitia* seropositivity in horses and donkeys in Israel, using IFAT cutoff titers of 1:64 and 1:256. Statistically significant results appear in bold.

Host	Parameter	Category	Number of Animals	Seropositive Animals, 1:64	*p* Value	Seropositive Animals, 1:256	*p* Value
Horses	Geographic location	North	160	5 (3.1%)	**0.009**	0	n/a
		Center	97	7 (7.2%)		0	
		South	90	12 (13.3%)		0	
	Sex	Geldings	171	12 (7.0%)	0.219	0	n/a
		Mares	166	10 (6%)		0	
		Stallions	10	2 (20%)		0	
	Breed	Mixed	163	11 (6.7%)	0.632	0	n/a
		Arab	47	5 (10.6%)		0	
		QH	69	5 (7.2%)		0	
		Other	68	3 (4.4%)			
Donkeys	Geographic location	Israel	49	41 (83.7%)	**<0.001**	28 (57.1%)	**<0.001**
		PA	49	13 (26.5%)		0	
	Sex	Male	60	34 (56.6%)	0.696	18 (30%)	0.694
		Female	38	20 (52.6%)		10 (26.3%)	
Both	Species	Horse	347	24 (6.9%)	**<0.001**	0	**<0.001**
		Donkey	98	54 (55.1%)		28 (28.6%)	
	Geographic location	North	160	5 (3.1%)	**<0.001**	0	**<0.001**
		Center	156	36 (23.1%)		14 (9%)	
		South	129	37 (28.7%)		14 (10.9%)	
	Sex	Male	241	48 (19.9%)	0.150	18 (7.5%)	0.267
		Female	204	30 (14.7%)		10 (4.9%)	

## Data Availability

All relevant data are included in the manuscript.
